# Understanding Immune Responses to Viruses—Do Underlying Th1/Th2 Cell Biases Predict Outcome?

**DOI:** 10.3390/v14071493

**Published:** 2022-07-08

**Authors:** Faith H. N. Howard, Amy Kwan, Natalie Winder, Amina Mughal, Cristal Collado-Rojas, Munitta Muthana

**Affiliations:** Department of Oncology and Metabolism, University of Sheffield, Sheffield S10 2RX, UK; amy.kwan@sheffield.ac.uk (A.K.); njwinder1@sheffield.ac.uk (N.W.); melody161@hotmail.co.uk (A.M.); cristal.colladorojas@gmail.com (C.C.-R.); m.muthana@sheffield.ac.uk (M.M.)

**Keywords:** viruses, T-cells, immunity, biomarkers, virotherapy

## Abstract

Emerging and re-emerging viral diseases have increased in number and geographical extent during the last decades. Examples include the current COVID-19 pandemic and the recent epidemics of the Chikungunya, Ebola, and Zika viruses. Immune responses to viruses have been well-characterised within the innate and adaptive immunity pathways with the outcome following viral infection predominantly attributed to properties of the virus and circumstances of the infection. Perhaps the belief that the immune system is often considered as a reactive component of host defence, springing into action when a threat is detected, has contributed to a poorer understanding of the inherent differences in an individual’s immune system in the absence of any pathology. In this review, we focus on how these host factors (age, ethnicity, underlying pathologies) may skew the T helper cell response, thereby influencing the outcome following viral infection but also whether we can use these inherent biases to predict patients at risk of a deviant response and apply strategies to avoid or overcome them.

## 1. Introduction

**Figure 1:**
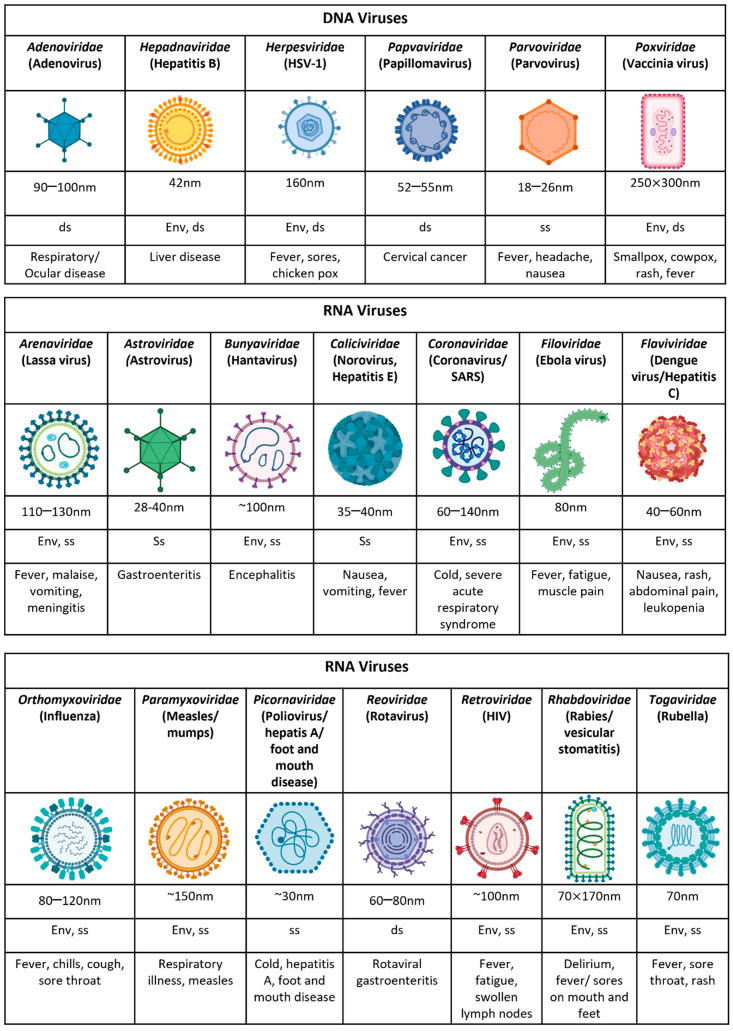
**The characteristic features of human viruses.** An overview of their typical size, genetic material, envelope, and their clinical presentation following infection. Env = enveloped, ss = single stranded, ds = double stranded.

Viruses are amongst the most diverse and abundant entities on the planet with the ability to convey genes from one organism to the next in order to develop, thrive, and survive (Figure 1). The clinical manifestation of viral infections is highly variable among individuals, with most suffering mild or subclinical infection, and others demonstrating severe and sometimes lethal disease. The ingenuity of the mechanisms these organisms employ for their survival and how they interact with our immunosurveillance systems are well-described and have resulted in effective vaccines for some (rubella, mumps, measles, etc.).

**Figure 2:**
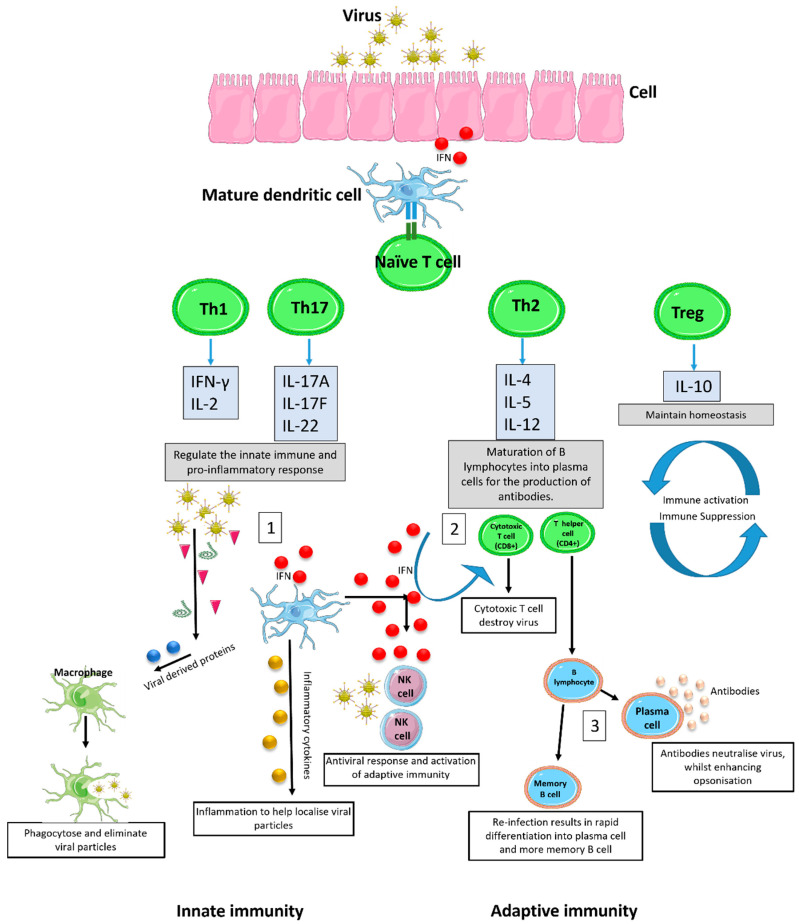
**Immune response to viral infection.** Viral infections elicit an immune response by first activating the innate immune system. Infected cells release IFN and pro-inflammatory cytokines that activate natural killer cells to destroy the viral infection. Simultaneously, infected cells express viral antigens on the cell surface, activating professional APCs such as dendritic cells (DCs). DCs interact with viral antigens through pattern recognition receptors for their maturation and in turn switch naïve T-cells into mature T-cells (Th1, Th17, Th2, and Tregs) that regulate both the innate and adaptive immune system. The innate response regulates a Th1 driven pro-inflammatory cascade, resulting in the recruitment of immune cells for rapid eradication of the infection (1). The adaptive immune system stimulates the differentiation and expansion of T lymphocytes into specific subsets, better known as cytotoxic T-cells (CTLs, CD8+) and T helper cells (CD4+). CTLs are responsible for the direct killing and eradication of viral particles and infected cells (2), whilst T helper cells recruit immune cells and stimulate the differentiation of B lymphocytes. B lymphocytes are responsible for the viral specific rapid response and long lasting immunological memory against recurring infection through the production of two subsets known as plasma cells and memory B-cells. Differentiation into plasma cell results in the production of virus specific antibodies for the neutralisation of viral progeny, the activation of the complement cascade, and antibody mediated opsonisation (3). Upon re-infection, memory B-cells stored within lymph nodes differentiate into active plasma cells, generating antibodies and the rapid activation of the adaptive immune system to provide effective relief faster than the first initial infection, usually leaving the individual asymptomatic. NK = natural killer cell. IFN = interferon.

The response to viral infection can be categorised into innate (non-specific defence mechanisms) and adaptive (specific defence mechanisms) immunity. The innate immune response is the first line of defence against viral infection and employs pattern recognition receptors such as Toll-like receptors (TLRs) for the detection of viral antigens or other viral components such as DNA or RNA [1]. Following detection, interferon (IFN) signalling proteins and pro-inflammatory cytokines are released from the infected cell into the surrounding environment, which in turn activate immune cells including professional antigen presenting cells (APCs) to stimulate the adaptive immune response (Figure 2) [2,3]. These specific immune defences employ B and T lymphocytes to help combat viral infection and develop long term immunological memory against recurring infections.

**Figure 3:**
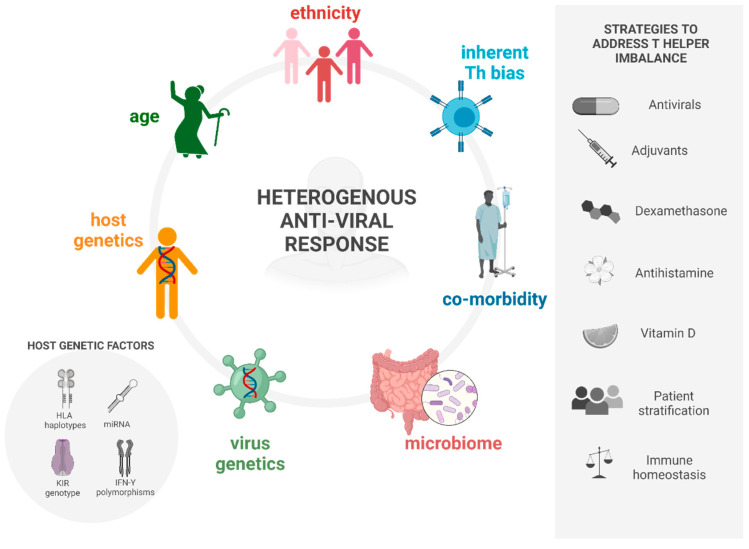
A schematic summarising the causes of a heterogenous anti-viral immune response and possible strategies to address T helper cell imbalance. Created with Biorender.

Simplistically, stimulation of the adaptive immune response via IFN and APCs allows for the expansion and differentiation of cytotoxic T-cells (CD8+) and naive T helper cells (Th0) into mature Th cells. There are four main Th subsets that have been identified and are involved in the immunological response to infectious disease (Figure 2). Arguably, the most important responses are Th1, primarily responsible for the pro-inflammatory response to intracellular parasites, and Th2, which is predominantly characterised by the production of antibodies [4,5]. The differentiation pathways of the Th1 and Th2 phenotypes are described by Kaiko et al. [6]. Despite well-characterised disease pathways and detailed immune responses (extensively discussed elsewhere [7,8,9,10]), an individual’s heterogeneous clinical response to viral infections that in turn govern whether a patient can mount an appropriate immune response to clear the viruses must therefore be due to host factors influencing the outcome. We hypothesize that these host factors specifically cause an imbalance in immune homeostasis, resulting in skewing towards a particular T helper cell response, which ultimately determines either disease progression or resolution. These include inherent immune activation biases, the age and ethnicity of individuals, and co-existing pathologies (Figure 3). Here, we review the current literature regarding the aetiology of these predispositions. Additionally, we summarise the strategies to modulate immune homeostasis and hence mitigate a deviant immune response to viral infection.

## 2. Causes of Deviant Immune Response in Viral Infections

Much focus has been given to the identification of specific human gene variants responsible for enhanced susceptibility or resistance to viral infection and it would be remiss of us not to include the genetic underpinnings that control viral infection outcomes; however, these have been reviewed elsewhere [11]. Briefly, the comparison of infected versus uninfected individuals have elucidated specific genetic factors responsible for divergent immune responses to specific viruses resulting in variability in both an individual’s susceptibility and outcome (examples in Table 1). These broadly fall into four categories: (1) Haplotypes of HLA important for encoding glycoproteins on the surface of nucleated cells for the presentation of antigen peptides to CD8+ lymphocytes with certain HLA alleles associated with protection [12,13]; (2) IFN-Υ3 polymorphisms alter the attachment of this cytokine to its receptor to modify the unspecific antiviral response; (3) miRNA is important in the replicative cycles of viruses and the deregulation of host miRNA can lead to increased cell survival, invasiveness, proliferation, and allow viral latency; (4) With a high tendency to suffer genetic mutations, killer-cell immunoglobulin-like receptors (KIR) and its role in cytotoxic T lymphocyte (CTL) efficiency heavily influences innate and adaptive immunity. Candidate genes responsible for identifying disease susceptibility loci such as those reviewed by Kenney et al. [11] are usually detected by large genetic screens. Interestingly, these investigations have also revealed changes in HLA allele frequencies and protective mutations according to ethnicity [14]. Additionally, it is known that infection rates increase in the elderly, which is attributed to an inability to mount an effective response and/or that this response becomes polarised as we age. This polarisation towards a particular T helper response is seen in the risk towards HCV infection [15], whilst the immune phenotype of a number of diseases have been identified as demonstrating Th bias (Table 1). Here, we discuss the evidence for the polarisation of the immune system by these host factors and how these disruptions in immune homeostasis impact viral immunity.

The most compelling evidence for differing clinical manifestations based on inherent predispositions can be sought from the use of pre-clinical models. The majority of viral pathogenesis studies are performed in inbred mouse models, which whilst cost-efficient and well-characterised, lack the diversity of responses seen in outbred populations such as humans, but the selection of particular strains may inadvertently bias the results. The most commonly used mouse models are ultimately known for their Th cell bias with Balb/c mice demonstrating a greater susceptibility to infection attributed to its Th2 bias whilst C57Bl/6 mice potentially benefit from a protective Th1 biased response. Whilst the selection of animal strain may simply be determined by their susceptibility to the infection in question, for example, the search for a small animal model of hepatitis E virus (HEV) determined rabbits to be a suitable model alternative to non-human primate and swine models following negative infection in Wistar rats, C57Bl/6, and Balb/c mice [36], the judicial use of particular animal strains is required, ideally with confirmation in multiple strains. Even then, this is not infallible, indeed, we have shown that predisposition to a particular Th cell response generated the opposite results. Inoculation with oncolytic HSV virus proved lethal in tumour-laden Balb/c mice but protective in C57Bl/6 mice [37], attributed to their prototypical Th bias. Conversely, the severity of encephalomyelitis induced by infection with Neuro-adapted Sindbis Virus (NSV) was fatal in C57Bl/6 mice, but Balb/c mice recovered [38]. Severe Ebola virus disease (EVD) has been linked to insufficient T-cell immunity with highly activated Th1 polarisation in tolerant animals but inhibition in lethal animals [39]. Here, the authors utilised the collaborative cross (CC), which is an octo-parental panel of reproducible inbred mice that comprise 90% of the genetic diversity across the entire laboratory Mus musculus genome, to further predict the EVD severity in mice by identifying transcriptional biomarkers, hence linking pathogenesis to host genetics. In these mouse studies, the outcomes following viral infection were induced by a Th imbalance, either as a pre-disposition through inbreeding or Th polarisation following stimulation. We focused on the reciprocal effect, in other words, how the host environment contributes to Th polarisation, thereby influencing infection outcome.

### 2.1. Heterosubtypic Immunity

In humans, does a Th cell imbalance precede clinical disease or is this a consequence of the disease process? The variability in the resting immune cell composition in mice has been dissected in 60 CC strains [40] and represents an important tool for analysing different immune cell frequencies and disease susceptibilities. Whilst in humans, we have a good understanding of the immune pathways involved following different viral stimuli and the shifts in Th cell balance, do these occur first or as a consequence of the disease process? Can we predict outcomes from the evaluation of immune cell compositions in a steady state? Olson et al. [41] conducted an investigation in humans by calculating the percentage Th1:Th2 cell ratios and suggested that Th bias may precede clinical disease. This may be a result of pre-existing immunity, the aetiology of which can be associated with exposure to related pathogens but also microbial communities associated with our microbiome regulate immune homeostasis [42]. It is thought that cross-reactive responses in the human population enhance the reaction of the immune system to an antigen that is related to one previously encountered. Additionally, T-cells mediate protection where no pre-existing antibodies are present, for example, in the context of a pandemic. However, the protective role of this heterosubtypic immunity is debatable with conflicting evidence for pre-existing CD4+, but not CD8+, T-cells associated with lower virus shedding and less severe illness in mouse models of influenza [43,44] whilst others have reported viral clearance mediated by antigen-specific CD8+ effector T-cells, with a role for CD4+ T-cells in maintaining CD8+ T and B-cell memory responses [45]. Additionally, early life dysbiota predisposes to inappropriate T-cell responses that are linked to compromised immune tolerance [46]. Indeed, an increased mortality to respiratory viral infection was reported following a reduction in diversity in the gastrointestinal tract associated with dysregulated Treg populations within the lung and intestine [47]. Bacteroides fragilis, in particular, enhances the induction of Th1 and Foxp3+ Treg cells and is thought to play a pivotal role in maintaining balance between inflammatory Th cells and suppressive Treg cells under healthy conditions [48]. In addition to bacteria, other viruses, fungi, and helminths can determine host immunity and virus infection. These trans-kingdom interactions described by Pfeiffer and Virgin [49] demonstrate both protective and detrimental outcomes. For example, the induction of an IFN response by helminths had a positive effect on respiratory viral infection whilst enteric helminths could enhance susceptibility to systemic viral infections that also had tropism for the GI tract [50]. Understanding how the macrobiome contributes to viral infection warrants further examination and may inspire new therapeutic approaches.

### 2.2. Ageing

It has been observed that a dysregulation of the adaptive immune system occurs in the ageing population. This change is two-fold: first the balance and effectiveness of T-cell types alters, and second, there is a weakened humoral response.

With regard to T-cells, there is conflicting evidence as to whether the absolute numbers of T-cells change with age. It is likely that T-cell absolute numbers stay somewhat similar, but confounders such as co-morbidities including cancer [51,52] and ethnicity may influence these results. In most of these studies, however, a shift towards less effective T helper cells is seen. This may be partly due to the involution of the thymus, resulting in decreased lymphoid cell numbers and also in naïve T-cell differentiation aberrances. With regard to the latter, the ratio between naïve T-cells and memory T-cells shifts with heavy memory T-cell balance, with the resultant effect being less naïve T-cells in circulation that may limit the immune response to new antigens in the ageing population [53]. Interestingly, a feature of these naïve T-cells is longevity. This is thought to be a resultant effect of the loss of thymic function. In thymectomised mice, naïve T-cells that develop longevity have demonstrated aberrant function with decreases in antigen and IL-2 cytokine production [54]. This is supported by Haynes et al., who also demonstrated that upon activation of aged naïve T-cells, less IL-2 is produced, which leads to poorly polarised T helper cells in vivo [55]. However, both authors also showed that if adequate IL-2 is provided to aged naïve CD4+ T helper cells in vitro, this dysfunction can be reversed. As IL-2 production is thought to be related to Th1 cells (Figure 1), one could speculate that ageing causes a shift towards a Th2 biased response, and indeed, there have been reports that suggest a shift in cytokine response towards a more Th2 predominance as we age [56], which may predispose individuals to other age related diseases such as cancer [57].

However, if this were the case, it would not explain the observation of poor antibody production following vaccination in the older population. Once polarised, Th2 cells activate B-cells to produce antibodies and eventually form B-cells. As CD4+ T-cells age, there is a reduction in this humoral response [58]. For example, the current pandemic has highlighted an age-dependent immune response to the Pfizer vaccine (BNT162b2). A cohort study with two age groups (vaccine receivers under 60 and over 80) compared antibody response after the 1st and 2nd dose of the vaccine. While the majority of participants in both groups produced specific IgG neutralising antibody titres, these were significantly lower in elderly participants [59]. Conversely, vaccine kinetics at later timepoints (>3 months) demonstrated comparable neutralising titres between the two groups (<50 vs. >55 years) [60]. Additionally, immune responses to a candidate vaccine against Herpes Zoster changed little with advancing age (50–59; 60–69; >70 years) 3 months post vaccination [61]. Whilst the SARS-CoV-2 study [60] did not analyse participants over 80 years, it highlights the need for studies on the durability of protection and the rational design of optimised and novel vaccines for the elderly [62].

This age-related decline in vaccine efficacy is also dependent on the germinal centre (GC) response involved in the affinity maturation of B-cells. Defects in the GC response have been extensively reviewed [63] and are largely driven by the perturbation of co-ordinated interactions between B-cells, T follicular helper (Tfh) cells, T follicular regulatory (Tfr) cells, and stromal cell subsets such as follicular dendritic cells (FDCs). For example, GC B-cells in aged mice lacked T-cell help as a result of a shift in the Tfr:Tfh ratio [64] attributed to poor T-cell priming [65] and the lack of mature antigen-specific Tfh cells [66]. Together with defects in the activation and expansion of FDCs [67], these contribute to an inferior GC response, resulting in poor memory B-cell formation and reduced antibody titres in older individuals [68]. The relationship between T-cells and B-cells may be mediated through the co-stimulatory molecule CD28, a molecule involved in T-cell activation, proliferation, and survival. During ageing, it is well-recognised that there is an increase in CD4+CD28- cells. These cells provide a predominantly Th1 response to infections in the elderly population and therefore may not activate the pathways, which result in antibody formation [69]. In support of the Th1 shift would be the clinical manifestation of certain traditionally “age related” diseases such as atherosclerosis. Th balance in atherosclerosis has been reviewed extensively, establishing it as a Th1 driven disease [70] with some indications that Th bias may predispose to its acquisition [71].

Nutrients have also been found to influence the Th balance, in particular, melatonin is known to have specific high-affinity binding sites on both Th1 and Th2 cells [72] but melatonin production is progressively reduced with age. Melatonin has the potential to enhance immune function in aged individuals and in patients in an immunocompromised state by stimulating the production of NK cells and CD4+ cells as well as the production and release of various cytokines from NK cells and T-helper lymphocytes [73]. Furthermore, when used as an adjunct to anti-viral treatment, melatonin enhances the survival of mice infected with influenza, which may be extrapolated to suggest that if we lose melatonin as we age, perhaps we also lose some of the efficacy of anti-viral therapy [74].

Anti-ageing drugs have been suggested as an approach to boost the effectiveness of COVID vaccines in older people. This includes using immunosuppressing drugs such as rapamycin (an approved mTOR inhibitor) and metformin (a type 2 diabetes drug). A small retrospective study in China found that the mortality among hospitalised diabetic patients with confirmed COVID-19 taking metformin was 2.9% compared with 12.3% in people who did not take the drug [75]. Interestingly, data on hospitalised individuals with COVID-19 with an average age of 75, some of whom were already taking metformin for obesity or diabetes, found a significant reduction in mortality among women taking metformin, but not among men [76].

Although T-cell changes in response to viruses with ageing is well-documented [77] (Table 1), whether these changes can be grouped into a Th bias remains unclear. Unfortunately, a recent analysis of COVID-19 vaccine trials found that the risk of exclusion of older adults is high [78], whilst the analysis of 18 recent trials revealed more than half had age cut-offs and many were at risk of excluding older participants, therefore data are still lacking in this area.

### 2.3. Ethnicity

Ethnicity has been defined as “the social group a person belongs to, and either identifies with or is identified with by others, as a result of a mix of cultural and other factors including language, diet, religion, ancestry and physical features traditionally associated with race” [79]. This is a rather broad term, and studies of ethnicity in viral response have the potential for many confounders such as social economic status and cultural norms that preclude social distance.

However, there have been longstanding observations of differences in the clinical progression of viral disease between differing ethnic groups, and viruses have been shown to evolve as a result of different immune responses in these different ethnic populations (Table 1). For example, the variation in disease severity with the hepatitis B virus has been extensively studied in the Chinese and Caucasian population and shows a greater prevalence of hepatitis B infection within the Chinese population [80], with this prevalence existing in spite of geographic location [81]. These observed differences may be due to inherent differences in immune profiles such as their T-cell repertoire, but not quantity, which was found to be significantly distinct between those of Chinese and Caucasian descent, with minimal cross over between the ethnic groups [82]. In this study, Tan et al. compared T-cell repertoires against the entire hepatitis B proteome in infected patients (47 of Chinese and 62 of Caucasian origin) and showed that these were divergent in the two ethnic groups with T-cell epitopes frequently found in Caucasian patients, but seldom detected in Chinese patients. The discordance demonstrates the ability of HLA polymorphisms to diversify T-cell responses, which has implications when considering therapeutic vaccine development. Here, hepatitis B-specific immune monitoring in Asian patients should not rely on the exclusive analysis of epitopes found in Caucasians or conform to algorithms that do not consider the clustering of different viral strains within different ethnic groups.

Another example is in HIV infection, where the rate of decline in CD4 count following HIV infection is a predictor of the progression to AIDS. In these patients, studies have shown a steep decline in patients of non-Afro-Caribbean descent [83]. To date, no study has investigated why this occurs and whether inherent Th biases may contribute to this observation. More recently, a lower CD4 T-cell count has been identified as a potential risk factor for severe COVID-19 infection for people living with HIV, irrespective of HIV virological suppression [84]. Increasing evidence supports a role for CD4 T-cells in the control and resolution of acute COVID-19 infection [85] and therefore any pre-existing CD4 T-cell depletion as described in patients with haematological malignancy [86] could be a potential barrier to COVID-19 immunity, hampering antiviral responses [87] and the development of immunological memory. More studies are required to determine the role of gender, race, and ethnicity, especially in areas of high HIV burden to help identify individuals who are particularly vulnerable to the impact of COVID-19 infection and need targeted vaccination interventions. For these patients, CD4 should be considered as a biomarker as patients may require tailored vaccine strategies to achieve long-term protective immunity.

Overwhelming evidence has highlighted that patients from ethnic minorities are disproportionately affected by COVID-19 compared to white individuals, with Asians at higher risk of intensive care admissions and death [88]. Whilst accumulating evidence supports a role for T-cells in SARS-CoV-2 (reviewed in Shroti et al. [89]), a complex picture is emerging, and data are yet to come. Additionally, dietary habits are an important modifiable risk factor for chronic inflammation with well-documented racial disparities in the prevalence of such diseases (cancer [90], diabetes [91], cardiovascular disease [92]). With various anti- and proinflammatory properties, studies have shown that Western-style diets rich in carbohydrates and fats are associated with higher inflammation compared with a Mediterranean diet of fruit and vegetables [93,94]. For example, the proinflammatory potential of diets consumed by African-Americans was significantly higher compared to Caucasian-Americans in a study of socio-economically disadvantaged women who were at high risk for exposure to COVID-19 in Alabama, USA [95]. Dietary nutrients can modulate the immune response directly on the function of immune cells or via the microbiome. Regulatory T-cells (Treg), in particular, respond to environmental cues and are important for maintaining immune homeostasis, as described by Arroyo Hornero et al. [96]. Th bias does feature in the development of autoimmune diseases and an ethnic disparity in prevalence and severity can be seen between the Afro-Caribbean and Caucasian populations. As autoimmune diseases are felt to be more prominent in those with a Th2 bias, T-helper cells and their ethnic difference have been explored within this population. In atopic dermatitis, an acute followed by chronic inflammatory condition of the skin, the pathology of the disease is characterised in Caucasians to show an initial Th2 biased approach followed by a Th1 bias in the chronic stages [97]. However, in the Afro-Caribbean population, a lack of the Th1 switch in the latter phases of disease is seen. In addition, a greater immune cell infiltrate, with a significant increase in atopic dendritic cells is seen in Afro-Caribbeans in comparison to Caucasians [98]. Perhaps in light of the ethnic variability observed with coronavirus [88], HIV [99], and dengue fever [100,101], namely an increased risk of infection and worse disease outcomes, Th bias in health and viral infection warrants further studies.

### 2.4. Co-Morbidities

Co-morbidities must be considered when evaluating immune responses for the control of viral infections. As the COVID-19 pandemic has highlighted, the risk of infection is significantly associated with conditions including diabetes [102], obesity [103], and respiratory conditions such as asthma [104] (Table 1). The immune phenotypes of a number of pathologies have been identified as displaying a Th cell bias (Table 2). This includes atherosclerosis, rheumatoid arthritis, type I diabetes, multiple sclerosis, and Parkinson’s disease, representing a Th1 immune phenotype, whilst asthma and Systemic lupus erythematosus represent a Th2 immune phenotype.

Patients with Parkinson’s disease (PD) demonstrate a Th1 biased immune signature brought about by reduced Th2, Th17, and Tregulatory cells (Treg), leading to a relative increase in Th1 cells as well as the preferential differentiation of naïve CD4+ cells towards the Th1 lineage from the in vitro functional testing of PD patient cells [114]. However, a group investigating the dysfunction of the peripheral immune system in PD in mice attributed the manifestation of the disease to a CD4+ T-cell differentiation bias towards Treg cells away from Th1 cells [115]. Age is the greatest risk factor for PD patients with the average age for diagnosis being 60 years, however, age is arguably associated with a trend towards Th2 bias.

Th1 cells have also been seen to drive atherosclerosis in mice [116] by the IFN-γ activation of macrophages and endothelial and smooth muscle cells, the inhibition of macrophage cholesterol efflux, and weakening the fibrous cap. Deficiency in IFN-γ or the Th1-differentiating transcription factor T-bet results in reduced lesion development [117]. Subjects from small clinical studies with coronary syndromes and advanced atherosclerosis were also found to have a bias towards Th1 [118,119,120]. A protective role for Th2 immunity has also been seen in cardiovascular disease [121]. Interestingly, in chronic inflammatory diseases such as rheumatoid arthritis, autoimmune encephalomyelitis as well as human alcoholic liver disease (ALD), a decline in Th1/Th2 responses has also been attributed to the upregulation and activation of Th17 cells [122]. This imbalance between Th17 and Th1 cells is also implicated in the pathogenesis of chronic hepatitis B infections [123]. Recent evidence suggests that viral infection may induce PD [124,125] and that cytomegalovirus (CMV) infectious burden was the main Th1 correlate in atherosclerosis [126]. Therefore, could the aetiology of the Th1 immune phenotype precede the clinical manifestation of these conditions? Either way, these studies suggest the importance of prospective studies of T-helper cell biasing.

Alterations in the Th1/Th2 ratio in cancer patients is a common feature of a malignant process and could result from the malfunction of Th1 cells, the activation of Th2 lymphocytes, or both [127]. A decrease in the Th1/Th2 ratio has been described in patients with glioblastoma [128], metastatic melanoma [129], non-Hodgkin’s lymphoma [130], breast cancer [131], head and neck cancer [132], and other tumour types. Conversely, a Th1 phenotype in breast cancer is associated with prolonged patient survival, whereby women with the highest ratios of Th1 cytokines to IL-5 levels were least likely to have aggressive phenotypes of breast cancer (oestrogen receptor negative [ER^−^] and triple negative breast cancer [TNBC]) [133]. Interestingly, the strongest associations were in premenopausal women, possibly indicating a more dominant role for immunosenescence and age-related biases driving the immune phenotype in postmenopausal women, although this is merely conjecture. These findings suggest that future therapies will need to address this profound Th2 skewing in order to have significant clinical efficacy in patients, possibly through vaccine strategies to shift tumour antigen-specific T-cell response to a more immunostimulatory Th1 bias [128].

Not only does the cancer itself demonstrate a distinct immune profile, but it is important to note that chemotherapies are, in essence, immunosuppressants and will alter an individual’s normal Th balance. The use of immune checkpoint inhibitors has provided a breakthrough in cancer therapeutics but may also help to sustain and enhance antiviral CD8+ T-cell responses by blocking the checkpoint programmed death ligand-1 (PD-L1) and IL-10, thereby generating the appropriate CD4+ Th1 help [134]. 

The cytopathic and immunostimulatory effects of viruses have been exploited for the generation of anticancer agents postulated as safer, more specific alternatives to traditional chemotherapies and radiotherapies. As the use of oncolytic virotherapy increases in the treatment of cancers following the FDA approval of T-Vec, an oncolytic HSV virus for the treatment of melanoma [135], do underlying Th biases including co-morbidities contribute to the variability in their efficacy? It is thought that CD8+ T-cell response is critical for the control of tumour growth and the co-expression of immunomodulatory transgenes stimulated an IL12 led Th1 response for enhanced survival in a mouse model of glioblastoma [136]. With this in mind, are asthmatic (Th2 immune phenotype) cancer patients more likely to experience side effects in response to virotherapy; conversely, would Parkinson’s patients require higher concentrations of virotherapy to demonstrate efficacy as their Th1 bias may contribute to faster clearance of the virus. Could we match oncolytic viruses to the immune phenotypes of these co-morbidities, in other words, use those that inhibit Th1 response (such as respiratory syncytial virus and influenza A) or titrate viral concentration against Th bias to individualise dosage? Another strategy could be the use of immunomodulators to support both immunotherapy efficacy but also deviant responses to viral infection, which we describe next.

## 3. Strategies to Address the Deviant Response

Manipulation of the Th1/Th2 balance has demonstrated therapeutic capabilities by reconstituting Th1 cells for the prevention of CD8+ T-cell decay thereby enhancing CD8+ T-cell activity and the control of infection of lymphocytic choriomeningitis virus (LCMV) [134]. Additionally, this idea of polarising the immune response to different arms of immunity has been proposed for the development of vaccines for the induction of tailor-made phenotypes for optimal protection against the influenza virus [137]. Additionally, rederivation of commonly used drugs including traditional antivirals, anti-histamines, aluminium salts (traditionally used as adjuvants), and vitamin D have demonstrated the ability to re-address the T-helper cell balance and may prove useful for deviant immune responses to viruses.

### 3.1. Antivirals

Most clinically approved antivirals used to treat viruses such as the human immunodeficient virus (HIV), herpes (B and C), and influenza (A and B) are designed to inhibit viral spread at various stages within the viral replication cycle without deactivating or killing the pathogen. Aside from this conventional use, growing evidence suggests that antivirals may also serve as a strategy to counteract and dampen the deviant response associated with viral infection in some patients. For example, the effects of Arbidol hydrochloride (ARB) on a wide spectrum of viruses including influenza virus, adenovirus, coxsackievirus, hepatitis C virus, and respiratory viruses have been previously reported [138,139]. In H1N1 (type A influenza) infected BALB/c mice, which usually develop lethal pneumonia, the administration of ARB suppressed disease mortality and viral load in the lungs in a dose-dependent manner [140,141]. These effects were mediated by the downregulation of the infection-induced cytokine inflammation profile (TNF-α, IFN-β, IL-1β, IL-6, and IL-12p40) and cytopathic effect, and the upregulation of IL-10, an anti-inflammatory cytokine, suggesting an alternative use for ARB post-viral infection.

Another agent known for its anti-viral inducing properties, IFN-α is a standard of care drug in the treatment of Behcet’s disease, an inflammatory blood vessel condition that displays a cytokine signature with a Th1-like resemblance [142]. In a double blinded clinical study, low-dose vaccination (150-IU) using IFN-α proved to reduce the severity of the respiratory symptoms caused by the influenza virus. Interestingly, such treatment demonstrated a statistically significant reduction in the incidence of acute respiratory illness, particularly in male study subjects aged 50 years or more, and those previously vaccinated against the 2009 seasonal influenza [143]. Additionally, the effects of IFN-α on purified memory T-cells (CD45RO+) isolated from peripheral blood mononuclear cells of patients with ongoing inflammatory conditions such as systemic lupus erythematosus and Behcet’s disease have also demonstrated a dampening of the Th1 pro-inflammatory response through the stimulated secretion of anti-inflammatory IL-10 [144]. Together, these data suggest that at low doses, IFN-α can diminish the pro-inflammatory response, possibly through the modulation of specific genes involved in the immune response. Given the current published evidence, the lack of appropriate strategies to manage virus-induced cytokine storms, and the fact that antivirals are relatively inexpensive and easily manufactured, this is a strategy worth considering, for example, in the management of global pandemics such as COVID-19, where the disease exhibits a dominant Th1 profile and where unwanted responses against viruses are major causes of mortality and morbidity rates.

Aside from antivirals, anti-fungals such as thimerosal have been shown to shift the immune response towards a Th2 bias (through increased production of IL-5 and IL-13), where they mediate the inhibition of proinflammatory molecules such as IL-1β, IL-6, IL-12p70, monocyte chemoattractant protein-1 (MCP-1), and IFN-Υ in human dendritic cells in vitro [145]. Furthermore, cannabidiol (CBD), a cannabinoid derived from Cannabis sativa, has been shown to promote a Th2-like immune response. In mice suffering from acute lung injury caused by lipopolysaccharide (LPS)-induced infection, a one-off CBD dose (20 mg/kg) administered prior to infection decreased the production of pro-inflammatory TNF, IL-6, MCP-1, and MIP-2 [146]. In human T-cells, Δ9-tetrahydrocannabinol (THC), another cannabinoid, reduced the production of IFN-α and increased the levels of Th2 cytokines (IL-4 and IL-10) in a concentration dependent manner after dendritic cell presentation of foreign antigens to T-cells [147]. THC is thought to interact with cannabinoid receptors on T-cells to suppress their activation, thereby dampening the inflammatory phenotype. The CB2 receptor is abundantly expressed in immune and immune-derived cells and its activation indirectly affects viral infections by altering host immune responses, particularly inflammation, along different signalling pathways. The anti-inflammatory and immunomodulatory activity of CB2 signalling in response to viral infections is comprehensively reviewed and suggests that the blockage of CB2 receptors is a potential target for the control of viral infection through the inhibition of immune suppressive effects [148].

### 3.2. Anti-Histamines

Another well-characterised category of drugs, anti-histamines, may also combat viral infections by influencing the way in which the virus enters the cells but without physically affecting the virus directly [149]. Anti-histamines are used to inhibit histamine production and enable mast cell stabilisation through alteration to the Th1/Th2 cytokine balance in basophils and T-cells whilst reducing chemotaxis, activation, and the survival of eosinophils as well as downregulating epithelial cell adhesion molecule expression [150,151,152]. Anti-histamines can play a huge role in combating chronic diseases such as atopic asthma as well as viral infections through changing Th1/Th2 homeostasis by increasing the stimulation of Th1 cells and the release of IL-2 and IFNγ cytokines whilst inhibiting Th2 activation, which in turn reduces eosinophilic inflammation and prevents airway hypersensitivity in mice [153]. Current studies investigating the potential for repurposing anti-histamines for the treatment of viral infections have identified two, carbinoxamine maleate (CAM) and S-(+)-chlorpheniramine maleate (SCM), following the screening of over 1000 FDA approved compounds [154] with potential antiviral activity. Both CAM and SCM inhibited the infectivity of influenza through blocking viral entry into the host cells during the early stage of the viral life cycle. However these effects were limited to viral endocytosis, as data suggest that CAM and SCM had no effect on viral attachment to the target cell, nor viral release upon lysis [154]. Similar studies have demonstrated the anti-viral effects of chlorcyclizine hydrochloride on hepatitis C viral infections by inhibiting HCV late-entry step before RNA replication and highlighting the potential of drug repurposing for effective anti-viral drugs [149]. Such studies highlight anti-histamines as good candidates for anti-viral treatments as they have excellent safety profiles from previous characterisations whilst being affordable.

### 3.3. Adjuvants

The promotion of specific adaptive responses to produce the most effective form of immunity may be assisted by the use of adjuvants. Typically used to increase the magnitude of an adaptive response to a vaccine, adjuvants are increasingly used to provide functionally appropriate types of immune response (e.g., Th1 cell versus Th2 cell, CD8^+^ versus CD4+ T-cells, specific antibody isotypes) and increase the generation of memory, especially T-cell memory [155].

For example, for insoluble aluminium salts (alum), the mechanism of adjuvanticity has been shown to activate the innate immune system via a Th2 response [156], induced by inflammatory dendritic cells through the endogenous danger signal, uric acid [157]. Additionally, Sharp et al. [158] demonstrated that mice administered with alum established a pro-inflammatory response hours after inoculation, showing increased release of interleukin-1β (IL-1β) 13, MCP-1, Eotaxin-1 (CCL11), histamine, and IL-5 [157,158]. In addition, alum have been known to initiate a robust antibody response linked to IL-4 secretion. Evidence shows that Gr1+ IL-4-producing cells increased B-cell proliferation and promoted an increase in the production of IgM antibodies, 24 h after inoculation with alum [159]. Such studies demonstrate that alum are essential for the recruitment and promotion of IL-4-producing cells, leading to a Th2 response and production of early IgM. Alum induced a particularly polarised Th2 cell response in mice with Th2 cell-dependent antibody isotypes to nearly all protein antigens whilst in humans, responses to proteins with alum tend to be a mix of Th2 and Th1 cells [160]. In contrast, more polarised Th1 cell responses are elicited by adjuvants that incorporate agonists of TLR3, TLR4, TLR7-TLR8, and TLR9 [161]. Complete Freund’s adjuvant (CFA) and CAF01 induced mixed Th1 and Th17 cell responses whilst MF59, ISCOMs, Toll-like receptor 2 (TLR2), and TLR5 ligands enhanced T-cell and antibody responses without altering their Th1/Th2 cell balance of the specific antigens [162]. 

The induction of certain cytokine repertoires by adjuvants has been shown to inhibit virus replication, namely hepatitis B [163] and enhance the immunity of antiviral vaccines including HIV [164] and influenza [165], with the latter demonstrating increased induction of T-helper type 1 (Th1)-biased cytokines. Therefore, the selection of an appropriate adjuvant is influenced by the type of CD4+ T-cell response required for protection but may represent an opportunity for prophylactic immunomodulation to support the use of combination immunotherapy.

### 3.4. Vitamin D

Vitamin D is a fat soluble steroid primarily known to help maintain healthy homeostasis in bone mineral density and general health with supplements administered to individuals at greater risk of osteoporosis and bone fractures [166]. Vitamin D has also been repurposed for the potential treatment of infection due to its influence on the non-classical immune response through the stimulation of the innate and adaptive immune response via a strong influence on the downstream gene expression of the inflammatory cascade. The pre-hormone 25-hydroxyvitamin D (25OHD) derided from vitamin D3 and its receptor vitamin D receptor (VDR) are activated by the enzyme CYP27B1, which is secreted by immune cells including dendritic cells, macrophages, monocytes, and respiratory epithelial cells [167]. Liu et al. showed that mycobacterium tuberculosis activation of macrophages via TLR2 induced the overexpression and upregulation of CYP27B1 and VDR [168]. This resulted in the formation of cathelicidin, a mycobacterial peptide responsible for the rapid elimination of tuberculosis infection through binding endotoxins and increasing the permeability of the bacterial membrane [169].

Vitamin D has also been linked to the eradication of viral infections, predominantly respiratory infections, most likely associated with a high expression of CYP27B1 by lung epithelia. Lung epithelial cells have been shown to reduce viral induction of inflammatory genes and increase the levels of the TLR co-receptor CD14 and cathelicidin upon treatment with vitamin D [167,170]. Several clinical studies have been conducted on vitamin D supplementation and their effect on respiratory infections, however, the results show conflicting data. Re-occurring respiratory infections in children showed a reduction in re-infections after six weeks of vitamin D supplements [171]. Similarly, an observational study that examined the relationship between the respiratory infections and serum levels of 25OHD demonstrated that low levels of serum 25OHD correlated with an increased rate of acute respiratory infections in Finnish males [172], children of Bangladeshi [173] and Indian [174] descent, and newborns [175]. In contrast, a large blinded, randomised, placebo-controlled trial of oral vitamin D3 with over 1500 elderly patients conducted in Britain showed no significant difference in the reduction in infection (type not specified) when patients were administered with vitamin D supplements [176]. These studies may also provide evidence for the role of ethnicity in an individual’s ability to resolve viral infections as a result of vitamin D deficiency whereby dark-skinned individuals with higher melanin levels will experience slower vitamin D synthesis to light-skinned individuals [177]. In addition, a study conducted predominantly on Caucasian patients demonstrated no significant effect on the incidence of recurring upper respiratory tract infections when patients were treated with a daily supplement of vitamin D over a 12 week period [178]. These studies showed that vitamin D may work more efficiently in children with recurring infections compared to the elderly, however these data demonstrated that the use of vitamin D in the fight against infections, primarily respiratory infection, may not be the most effective as a lone treatment due to inconsistencies found within the literature. Data such as these suggest that vitamin D is an important regulator in the inflammatory response within infected individuals, and that vitamin D deficiency may lead to dysregulation of this delicate response [179].

Vitamin D can also influence the adaptive immune system, particularly T lymphocyte regulation via the upregulation of Th2 cytokines associated with an anti-inflammatory response, whilst simultaneously stimulating the differentiation and expansion of regulatory T-cells through VDR activation. Mechanisms for vitamin D induced antiviral activity are well-described [180,181], however, deciphering these diverse biological activities in the context of different viral infections requires further investigation including validated markers of immune modulation [182].

### 3.5. Dexamethasone

Dexamethasone is a corticosteroid affecting the hypothalamic–pituitary–adrenal axis (HPA) for the regulation of metabolism, development, homeostasis, and cognition [183]. It targets inflammation by binding to the glucocorticoid receptor (GR) on the cell membrane, influencing translocation, and promoting immunosuppression by preventing the extension of the cytokine storm [184]. This provides a rapid relief of inflammation and hence its use extends to the treatment of rheumatoid arthritis [185], severe allergies, and skin diseases, to cancers such as lymphoma [186]. Dexamethasone has been applied as a pioneering treatment in reducing inflammatory effects by rebalancing the innate and adaptive immune response. The migration of immune cells to the site of inflammation causes the activation of deviant immune responses [184]. Thus, dexamethasone can be used to prevent the persistence and maintenance of the immune system [187]. In a study with intracranial leukemic tumours, clear Th2 domination occurred. Co-stimulation with dexamethasone blocked naïve T-cell proliferation and impaired the maturation of T-cells via the upregulation of CTLA-4 mRNA and protein in CD4+ and CD8+ T-cells. This also affected the memory T-cells due to the partial immunological rescue in dexamethasone and CTLA-4 blockade treated tumour-bearing mice and enhanced survival [188]. Therefore, there are alterations in the Th ratio that must be due to persistence of the immune response.

The Th bias can be seen to influence disease severity. Some studies in mice have shown that Th cells lose the ability to skew towards Th2, which prevents the secretion of pro-inflammatory cytokines [189]. A shift towards Th2 immunity in diseases such as AIDS increases the risk of immunosuppression, but treatment with dexamethasone has been seen to rebalance this [190]. However, in rheumatoid arthritis, this balance is skewed towards Th1 cells and cytokines such as TNFα and IFN-γ [191]. Interestingly, the innate immune response is also supressed. Studies suggesting that NK cells interact with Th cells have demonstrated that dexamethasone affects cytokine engagement with receptors, but does not alter the expression of receptors in Stat4 knockout mice [192]. In addition, pre-treatment with dexamethasone does not reduce IL-4 and IL-12 expression, but responsiveness to receptors and phosphorylation is prevented. Therefore, this suggests that inhibiting the phosphorylation of these molecules affects NK cell signalling with Th1 cells but not Th2.

Studies during the coronavirus pandemic have allowed for the significant use of dexamethasone and have shown promising results, especially in decreasing the production of pro-inflammatory cytokines such as IL-1, IL-6 [193], and IL-17, and upregulating anti-inflammatory cytokines [194]. These pro-inflammatory cytokines have been linked to the severity of COVID-19 and led to the imbalance of Th cells towards Th2, for example, delaying Th1 response interferons [195]. A study conducted in Wuhan, China showed that the initial infection led to Th1 responses and disease progression led to Th2 responses, with cytokine release and cytokine storm [196]. However, further study is needed to determine pathogenesis. On the other hand, blood samples of COVID patients showed 100% CD4+ T-cell responses and the secretion of IFN-γ with Th1 polarisation [193]. The treatment of COVID-19 with dexamethasone revealed that it was able to combat severe disease presentation. Patients in the largest randomised controlled RECOVERY trial showed a 28-day reduced mortality and was especially effective in reducing mortality in those that required mechanical ventilation or presented with a longer duration of symptoms [197], however, there was no data on the level of oxygen support. Similarly, the efficacy of dexamethasone in patients recruited globally in a meta-analysis also showed a reduced 28-day mortality in severe COVID patients [198]. Another study in Spanish patients that were randomly assigned to receive intravenous injections of dexamethasone had higher ventilator-free days (VFD) compared to the control group [199], however, these results were not significantly different possibly due to the low patient enrolment. Although it has been recommended as the standard of care for the moderate dosage of dexamethasone in patients with acute respiratory distress syndrome (ARDS) from COVID-19 because of the risk of sepsis and cytokine storm complications [200], the usage of dexamethasone must be carefully considered due to its ability to induce B-cell-mediated antibody production [201], the protective function of T-cells [202], and macrophage-mediated clearance of apoptotic cells [203], causing a higher plasma viral load and introducing the risk of secondary infections. Equally, little research has been conducted on its interaction with other antivirals and lengthy use throughout treatment, for example, in the ICU, but still provides a therapeutic benefit as it is cheap and accessible. However, the Th bias in treatment with dexamethasone is still conflicting and whether systemic administration is beneficial for the immune system in the long-term.

### 3.6. Patient Stratification

Stratifying patient populations is an important tool to identify risk factors and predict outcomes. Recent examples have utilised human leukocyte antigen (HLA) and killer cell immunoglobulin-like receptor (KIR) genotyping to stratify the risk of CMV infection in kidney transplant patients [204] and predict the outcome of hepatitis B virus (HBV) infection [205] as well as identify the risk factors involved in acute viral encephalitis following HSV infection [206,207]. The number of co-morbidities associated with a Th biased immune phenotype that contributes to an increased COVID-19 infection risk suggests a role for Th imbalance in the outcome following viral infection and provides grounds for stratifying treatment protocols to these patients based on their Th status. How this information is used, however, requires more investigation, for example, if an efficient adaptive anti-viral immune response is thought to be of the Th1 type, do individuals with an underlying Th1 condition clear viral infection quicker, or are they more susceptible to viruses that can inhibit Th1 response by downregulating IFNs?

## 4. Discussion and Concluding Remarks

Further studies investigating Th bias in humans have not been performed as far as we are aware, but Huang et al. noted that patients infected with COVID-19 in Wuhan, China (*n* = 41) had high amounts of IL1B, IFNγ, IP10, and MCP1 (detected in serum by PCR), probably leading to Th1 cell response. However, infection also initiated increased secretion of T-helper-2 (Th2) cytokines (e.g., IL4 and IL10) that suppress inflammation [196]. Additionally, we have shown that the induction of a Th1 response following the administration of oncolytic Herpes Simplex Virus-1 was well-tolerated and provoked an anti-tumour immune response in a clinical Phase I/IIa trial of mesothelioma [208] as well as mouse models involving prototypical Th1 biased mice [37], which is in support of our hypothesis that host Th bias plays a large role in the outcome following viral infection. Additionally, in this context, it may also have the potential as a predictive biomarker for clinical response to virotherapy. Here, we presented evidence of skewing of the immune phenotype prior to viral infection attributed to ethnicity, age, pathologies, and microbiome (Figure 3). Together, they provide the justification for the Th profiling of patients as part of a diagnostic health screen. Stratifying patient populations is an important tool to identify risk factors and predict outcomes. Recent examples have utilised human leukocyte antigen (HLA) and killer cell immunoglobulin-like receptor (KIR) genotyping to stratify the risk of CMV infection in kidney transplant patients [204] and predict the outcome of hepatitis B virus (HBV) infection [205] as well as identify risk factors involved in acute viral encephalitis following HSV infection [206,207]. The number of co-morbidities associated with a Th biased immune phenotype that contribute to an increased COVID-19 infection risk suggests a role for Th imbalance in the outcome following viral infection and provides grounds for stratifying treatment protocols to these patients based on their Th status. Additionally, the belief in a Th1/Th2 dichotomy oversimplifies the dynamic cytokine microenvironment controlling cell fate, making the selection of a traditional cytokine panel for such discriminations difficult and open to interpretation, so may therefore require a multi-omics approach. Practically, this emerging understanding of determinants of biased Th immune phenotypes could provide opportunities to effectively exploit them for the therapeutic purposes as described. Giving the body systemic support to maintain immune homeostasis rather than deploying tailored magic immunotherapeutics may prove to be the most effective strategy for the resolution of viral infections.

## Figures and Tables

**Table 1:** Evidence of the influence of host features on viral infection. TNF = tumour necrosis factor; DHF = dengue hemorrhagic fever; HCV = hepatitis C virus; RSV = human respiratory syncytial virus; IAV = influenza A virus; WNV = West Nile virus; IFN = interferon; DC = dendritic cells; ACE2 = angiotensin-converting enzyme; Nab = neutralising antibody; HRV = human rhinovirus.

Feature	Virus	Effect on Host	Refs.
**Host genetic susceptibility**			
TNF-α (−308) GG genotype IL-10 (-592/-819/-1082) CCA/ATA genotype IL-10 (-592/-819/-1082) ATA/ATG genotype	Dengue	Development of severe dengue in Sri Lankan patients. Risk factor to developing DHF. Protective factor from DHF.	[16]
*G6PD* gene	Dengue, coronavirus, enterovirus	Deficiency enhances viral infection.	[17,18,19]
A117V polymorphism in the NS2A	Zika	Increased virulence by reducing host innate immune responses and viral-induced apoptosis in vitro.	[20]
HLA DRB1*11	HCV	Protects from disease progression.	[21]
HLA*0405, HLA DRB1*0301, DQB1*0201	HCV	Viral persistence and chronic infection.	[22]
*IFIH1*	RSV	Deficient individuals unable to induce IFN-β, rendering them susceptible to infection.	[23]
Q421X	IAV	Impaired IFN-α production causes life-threatening condition.	[24]
**Ageing**			
Axl, Mertk	WNV	Age-related upregulation of regulatory receptors facilitates viral uptake by increasing blood–brain barrier permeability.	[25]
T-cell defects	WNV	Insufficient number and quality of effector antiviral T-cells underlie age-related susceptibility to WNV.	[26]
Histone modifications	IAV	Age-associated altered histone expression decreases IFN production by myeloid DCs.	[27]
miR-181a deficiency in T-cells	WNV	Hallmark of ageing. Impairs T-cell expansion, viral clearance, and recall response.	[28]
**Ethnicity**			
IFN	HCV	IFN effectiveness in blocking viral production significantly greater in White versus African-American patients.	[29]
ACE2	COVID-19	ACE2 (receptor for cellular entry) expression significantly higher among Asians compared to African-Americans and Caucasians.	[30]
Nab	Rubella	Individuals of African descent have significantly higher rubella-specific NAb levels than European or Hispanic individuals.	[31]
**Co-morbidities**			
Obesity	Influenza H1N1	Decreased CD8+ T-cell activation results in inability to mount protective immune response	[32]
Asthma	IAV	Increased susceptibility to heterologous secondary influenza due to defective mucosal antibody responses.	[33]
Cancer (melanoma/RCC)		Tumour antigen-specific Th2-type polarisation of CD4^+^ T-cell responses in the peripheral blood of patients with RCC or melanoma.	[34]
Type 2 airway disorders (allergic asthma, allergic rhinitis, CRSwNP)	HRV16	Type 2 cytokines increase susceptibility to viral infection in airways via changing the epithelial structure and production of interferons. A Th2 bias induces a deficit in defending the mucosa against viral and bacterial infections.	[35]

**Table 2:** Th-dependent diseases. IFN = interferon; IL = interleukin; TNF = tumour necrosis factor; CNS = central nervous system; Treg = T regulatory cells; PD = Parkinson’s disease.

	Evidence for Th Polarisation	Ref.
**Th1**		
Atherosclerosis	CD4+ T-cells dominate atherosclerotic plaques. Increase IFN-γ and IL-2, IL-12, IL-18.	[70]
Rheumatoid arthritis	Increase in IFN-γ+CD4+ T-cells in peripheral blood and IFN-γ and TNF-α expression. Reduction in IL-6 and IL10 expression.	[105]
Type I diabetes	High IFN-γ expression drives persistent signal in pancreatic beta cells.	[106]
Multiple sclerosis	IFN-γ-producing Th1 cells most frequent Th cell subset in the CNS.	[107]
Parkinson’s	PD patients more Th1 cells and fewer Treg cells. CD4+ T-cells mediate brain inflammation.	[108] [109]
**Th2**		
Asthma	Production of Th2 cytokines IL-4, IL-13, IL-5, increased production of IgE by B-cells. Genes that enhanced Th2 polarisation (IL17RB) and Th2 cytokine (IL-25) production were upregulated in asthma.	[110] [111]
Ulcerative colitis	Overexpression of Bcl2L12 by CD4+ T-cells upregulates Th2 responses and downregulates Th2 ell apoptosis.	[112]
Chronic fatigue syndrome	Shift from Th1 to Th2 profile correlated with illness parameters including increase in IL-4 and reduced natural killer cell cytotoxicity.	[113]
